# Electrical Stimulation and Cellular Behaviors in Electric Field in Biomedical Research

**DOI:** 10.3390/ma15010165

**Published:** 2021-12-27

**Authors:** Shiyun Meng, Mahmoud Rouabhia, Ze Zhang

**Affiliations:** 1 Department of Materials Science and Engineering, College of Environment and Resources, Chongqing Technology and Business University, Chongqing 400067, China; shiyun_meng@hotmail.com; 2 Département de Chirurgie, Faculté de Médecine, Axe Médecine Régénératrice, CHU de Québec—Université Laval, Quebec, QC G1V 0A6, Canada; 3 Groupe de Recherche en Écologie Buccale, Faculté de Médecine Dentaire, Université Laval, Quebec, QC G1V 0A6, Canada; mahmoud.rouabhia@fmd.ulaval.ca

**Keywords:** biophysical stimuli, electrical stimulation, electrode, conductive polymer, in vitro

## Abstract

Research on the cellular response to electrical stimulation (ES) and its mechanisms focusing on potential clinic applications has been quietly intensified recently. However, the unconventional nature of this methodology has fertilized a great variety of techniques that make the interpretation and comparison of experimental outcomes complicated. This work reviews more than a hundred publications identified mostly from Medline, categorizes the techniques, and comments on their merits and weaknesses. Electrode-based ES, conductive substrate-mediated ES, and noninvasive stimulation are the three principal categories used in biomedical research and clinic. ES has been found to enhance cell proliferation, growth, migration, and stem cell differentiation, showing an important potential in manipulating cellular activities in both normal and pathological conditions. However, inappropriate parameters or setup can have negative effects. The complexity of the delivered electric signals depends on how they are generated and in what form. It is also difficult to equate one set of parameters with another. Mechanistic studies are rare and badly needed. Even so, ES in combination with advanced materials and nanotechnology is developing a strong footing in biomedical research and regenerative medicine.

## 1. Introduction

After Italian physician Luigi Galvani first noticed the leg movement of brain-dead frogs following an exposure to electricity, the electrical properties of tissues and cells have been extensively studied, leading to the formation and maturity of the entire domain of electrophysiology. Now, the study of electrical properties of tissues and cultured cells in laboratories has become routine. For example, patch-clamp techniques are very useful to study ion channels in cell membranes, and electroporation is a powerful tool to deliver large cargo to the inside of cells by increasing membrane permeability. The monitoring of and intervention to heart and brain electrical activities through external or implantable electrodes are now essential in medicine.

While electrophysiology is a highly developed area with a long history, electroactivity as an intrinsic property of human tissues and an important phenomenon in tissue regeneration and wound healing has not been widely recognized among material scientists. This is clearly testified by the reported biomaterials that are mostly insulators, and by the large absence of electricity as a tool in regenerative medicine.

Communication between cells is known through fixed and soluble mediators in various forms, such as gas, ions, peptides, and proteins. A stable match between an epitope and a paratope, in antibody-antigen recognitions, only happens when they encounter each other at a sufficient proximity after diffusing through the charged extracellular matrix (ECM) and competing successfully with solvent molecules. As all players in this recognition process, including ECM, solvent molecules, antibodies, and antigen-presenting entities, are sensitive to electric field (EF), owing to the charges and polar groups in their molecules, the distribution and movement of these players, among other properties, can be manipulated or influenced by either endogenous or exogenous EF, theoretically at least, leading to the electrical modulation of cellular interactions. In fact, various electrical stimulation (ES) modalities have been proven effective in regulating cellular activities such as ion transportation and gene expression [1], production of proteins and intracellular reactive oxygen species [2], secretion of chemokines and cytokines, neurite growth, and cell migration, etc. [3,4,5]. All of these observations suggest that a physiologically relevant endogenous or exogenous ES may serve as an effective tool to affect or regulate cellular and tissue homeostasis [6,7,8].

Applying an electric or electromagnetic field to cultured cells or the human body requires specialized equipment; such equipment is usually non-standardized and not readily available. In literature, ES stimulation to mammalian cells are reported in a very diverse way: some of them are based on cell types, and others are based on the stimulation methods or the characteristics of EF such as direct current (DC) vs. alternative current (AC), or the materials used to mediate ES. As we feel methods are the key to perform such studies, this review is organized according to the experimental setups and the materials used to mediate ES.

## 2. Electrode-Based ES

Electrodes are the most popular tools to apply an ES to cells or tissues with, likely due to its simplicity. People use electrodes in different ways, and the data are hardly comparable. We grouped the techniques into the following three categories according to how EF is established.

### 2.1. EF Between Two Electrodes Immersed in Culture Medium

**Figure 1:**
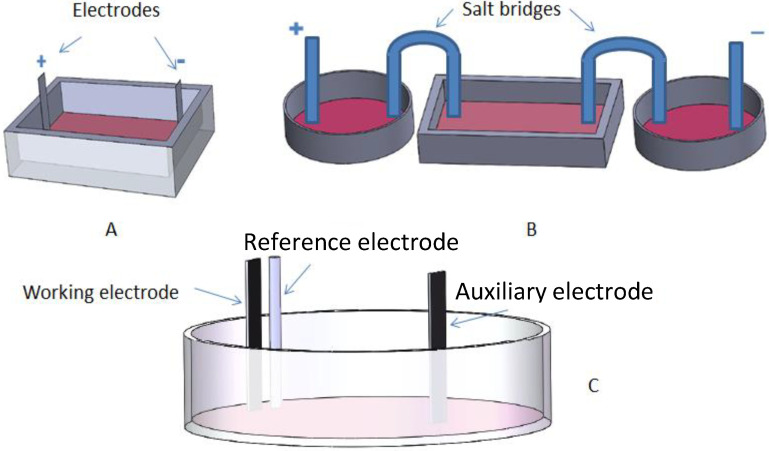
(**A**) EF between two electrodes immersed in cell culture, in which the electrodes may take different forms such as L and against the bottom to make the EF more uniform; (**B**) Using salt bridges between electrodes to avoid the diffusion of electrochemical reaction products into culture medium; (**C**) Working electrodes as a cell culture substrate in a three-electrode system, where the working electrode can be a metal, a glass slide coated with indium tin oxide, or a conducting polymer.

In this approach, electrodes are directly immersed in the culture medium of a cell culture plate (Figure 1A). To avoid erosion, many researchers used noble metals or graphite as electrode, including platinum (Pt) (1.19 E° (V), reduction potential), gold (Au) (1.83 E° (V)), titanium (Ti) (0.72 E° (V)), silver (Ag) (0.80 E° (V)), and other alloys [9]. In a two-electrode system, the two electrodes, i.e., anode and cathode, are normally in direct contact with the culture medium or tissue, with an EF established between the electrodes through electrolyte (culture medium). Such a system only needs two electrodes and a power source. Cells are cultured either in a normal Petri dish or in home-made culture chambers. A three-electrode system may also be used where cells directly adhere to the working electrode, which will be discussed in Section 2.3. Caution must be taken if Faradaic current exists as redox products may be cytotoxic and interfere with cellular activities even at a very low concentration. Uniformity of EF to the cells at different locations and the possibility of electrophoresis are two other factors that must be considered as well.

With a home-made device, Altamirano et al. [10] used electrodes made of platinum or silver wires to stimulate nerve cells at low frequencies of 2 to 3 Hz. They found that such stimulations caused rapid “fatigue” of transmission that didn’t occur at high frequencies. In another early work, a very low level of electric current ranging from 0.00115 to 11.5 nA/mm^2^ was delivered through two platinum wires bathed in the incubation medium to chick embryo trigeminal ganglia for different times, varied from 2 to 96 h, showing an enhanced outgrowth of nerve fibers, a higher neuron survival rate, and the cell migration towards the cathode [11]. Aro et al. [12] inserted two Pt-Ir electrodes of 0.5 mm diameter in flasks where rat fracture callus cells and human peripheral blood lymphocytes were cultured, respectively. The maximal ES current density was 10.6 μA/mm^2^. Their results showed that ES affected the uptake of DNA precursors in both cell types at different phases of cell proliferation. Moreover, they concluded that electric current and chemical mitogens might have affected lymphoid cells in similar or identical mechanisms.

Strong EF and electrochemical products also damage cells and tissues. Placing a pair of parallel silver wires in a Petri dish to deliver current to the cultured neuroblastoma cells (mouse and human), Krauthamer et al. [13] reported cell lysis and damage to neuritis depending on the tEF intensity. While the authors didn’t measure the silver-ion concentration in the medium, they claimed that the medium exposed to ES was used to culture other cells without showing any damage. Lyte et al. [14] immersed platinum wires 1 cm long and 0.2 mm in diameter as electrodes in an RPMI-1640 medium to study the effect of ES on lymphoma cell growth. They reported that ES within a narrow range of weak DC, i.e., 0.1 to 10 µA, increased EL4 cell growth; however, ES at approximately 17 μA reduced the growth of the lymphoma cells. Berger et al. [15] placed two graphite electrodes in parallel immersed in medium inside a standard culture flask, which supplied a current density of 15 mA/cm^2^ at a voltage gradient of 0.5 V/cm and frequencies from 0.1 to 7.0 Hz. They reported myocyte contractions at physiological frequencies induced by a continuous ES in a short-term culture of the primary myocytes. The authors observed no pH change greater than ±0.10 over a 24 h period. In 1996, Fagerheim et al. [16] exposed human polymorphonuclear neutrophils to electric current in a high voltage gel electrophoresis apparatus where the resistance in the apparatus could be regulated by varying the number of layers of aluminum foil and gel. They observed free oxygen radicals generated from broken deoxyribose and methional when the current intensity became 25 to 40 nA/cm^2^ at 24 or 40 V/cm for 1 h. Hinsenkamp et al. [17] cultured skin keratinocytes on decellularized dermis at the air–liquid interface, then used a biphasic, asymmetric and charge-balanced current applied through Pt electrodes at a frequency of 40 Hz and a pulse width of 0.25 ms to stimulate the cells. At a current intensity of 20 mA, the stimulation was in the form of trains of bursts, with each train lasting 4 s, followed by a 4 s pause for 40 min/day in 11 days. They showed that the migration, proliferation, and differentiation in the electrically treated samples were significantly and favorably modified.

Through an array of stainless steel electrodes, Janigro et al. [18] used AC at 50 Hz to reduce the proliferation of different human and rodent tumor cell lines (C6, HT-1080, H-1299, SKOV-3, and PC-3), reporting that the ES impacted the cellular process by affecting potassium channels. Wang et al. [19] delivered ultra-short (4 ns) and very high strength (10 to 80 kV/cm) pulses to the micro-chamber electrodes. The micro-chamber was a rectangular channel that was 100 mm wide, 30 mm deep, and 12 mm long, with gold-plated walls as electrodes that were micro-fabricated with photolithographic method on glass microscope slides. They reported that the ES induced cardiac cell excitations by mobilizing intracellular Ca^2+^ and creating transient nanopores in the plasmalemma. Kim et al. [20] designed a bioreactor composed of a biocompatible cylindrical Teflon body containing a flexible polyimide electrode and an implantable stimulator made of two rectangular gold patterns. The human mesenchymal stem cells (hMSCs) seeded on a collagen sponge in the bioreactor were stimulated with a biphasic current of 20 to 40 µA for 100 µs at 100 Hz. They reported that both the cell proliferation and expression of type I collagen increased significantly. Vunjak-Novakovic et al. [21] generated a linear EF between two rod-shaped electrodes 1 cm apart in a Petri dish. They tested different electrode materials, including graphite, titanium, stainless steel, and titanium-nitride coated titanium. They also tried to optimize the parameters, i.e., amplitude from 1 to 6 V/cm, duration from 0.25 to 10 ms, and frequency at 1, 3, and 5 Hz. After culturing neonatal rat ventricular myocytes under the ES, they reported that the engineered cardiac tissues stimulated at 3 V/cm and 3 Hz recorded the highest tissue density, the highest concentrations of cardiac troponin-I and connexin-43, and the best contractile behavior.

Direct ES through electrodes in culture medium is the simplest strategy. However, depending on the type of metals used, an irreversible metal dissolution might occur at an unacceptable rate that may modify the pH value of the medium. In addition, EF may cause a gradient in ion distribution in culture medium that could differentially affect cell physiology and increase the difficulty of discriminating the specific cell responses due to ES. Most importantly, a constant current generates electrochemical products at electrode/medium interface, which could harm the cells. From these considerations, the charge-balanced pulsed ES could be a solution [22].

### 2.2. Using Salt Bridges between Electrodes

By filling U-shaped glass tubes with a solution made of a high concentration of inert electrolytes such as KCl or KNO_3_ and gelling agents such as agar or gelatin, salt bridges allow the movement of ions to transport charges while blocking the diffusion of other substances. Instead of directly immersing electrodes in a culture medium, the purpose of using salt bridges is to prevent contamination to the culture medium by the redox products generated at electrode/liquid interface. This design is a better alternative to the direct ES with metal electrodes mentioned in the previous text. The downside is the complexity of the equipment that makes multiple parallel experiments difficult. Under this configuration, the actual potential gradient in the culture medium should be measured between the ends of the two salt bridges immersed in the culture medium (Figure 1B).

In 1988, Kaplan et al. [23] reported that direct current ES could influence the formation of neuronal aggregates by promoting neuronal survival at a current density of 15 nA/cm^2^; however, the authors mentioned that current densities less than 1 nA/cm^2^ or greater than 150 nA/cm^2^ might be detrimental to neuronal cell survival. In their experimental design, they modified 60 mm coupled Petri dishes as the stimulation units. They used platinum alloy as working electrodes with 2% agar salt bridge placed between the anode and cathode chambers [23]. Using salt bridges filled with 3% agar in a minimum essential medium (MEM) to connect Ag/AgCl electrodes, Ferrier et al. [24] investigated the effects of 0.1 and 1.0 V/mm EF on primary rabbit osteoclasts and rat osteoblast-like cells. The authors reported that the primary osteoclasts migrated rapidly toward the positive electrode, whereas the osteoblast-like cells migrated in the opposite direction toward the negative electrode. However, an anodal movement of cells at a speed of ~7 μm/h in EFs between 100 and 450 V/m was reported in 2019 [25]. Harris et al. [26] designed a thin strip of channel in a Petri dish to study the reorientation of fibroblasts in various electric voltages. They reported that the cells elongated perpendicular to the EF and strengthened their contractility in that axis. In their design, the electric current was introduced through a pair of salt bridges filled with agarose gel in saline. In a similar way, Krauthamer et al. [13] designed a closed-cell culture chamber with coverslips and silicone grease. The salts generated by the electrodes, a pair of 0.5 mm silver-wire, were excluded by the agar-salt bridges connecting the cell culture chamber and the electrodes. They reported that both human and mouse neuroblastoma cells were lysed or their neurites damaged at 10 and 3–5 V/cm, respectively. McCaig’s group has studied extensively the cellular behaviors in EF using salt bridges. They reported that the exposure to a physiological EF (200–250 mV/mm) significantly modified the orientation and the migration rate of epithelial cells. In their experiment setup, agar-salt bridges were used to connect the silver/silver chloride electrodes and the culture pool, to prevent the diffusion of any electrochemical products from electrodes into the cultures [27,28]. Sun et al. [29] characterized the effects of ES on the adhesion and orientation of bone marrow-derived mesenchymal stem cells and fibroblasts seeded in a 3D collagen scaffold/gel. The scaffold was in a custom-designed chamber where electric current from a power supply was applied through 2% agar salt bridges to avoid unwanted byproducts from metal electrodes and minimize the culture’s pH change. They found an optimal EF-regulated cell shape that was cell-type dependent and influenced cell orientation in the process of cell differentiation and growth. Meng et al. [30] glued glass strips with silicone grease to build a transparent cell culture chamber and then introduced ES in the form of direct current (DC) through calomel electrodes and salt bridges. When neural progenitor cells (NPCs) were exposed to a range of physiological EFs, they showed a highly directed migration towards the cathode. With a similar ES device, Borgens et al. [31] concluded that some cells such as chick root ganglia neurons and sympathetic neurons oriented perpendicularly to the lines of EF in a manner similar to hippocampal cells. Nguyen et al. [32] reported that Schwann cells showed a greater alignment perpendicular to the EF at higher current densities (106 mA cm^−2^ at 245 mV mm^−1^), and reached the maximum alignment within 8 h. On the other hand, alternating current (AC) at 2–1000 Hz did not cause cells to reorient but shaped them into round, flat and large cells that showed more processes than the control or the DC stimulated cells. Their design used two long strips of polydimethylsiloxane (PDMS) placed above the channel to isolate the medium on opposite sides of the channel. Bayat et al. [33] delivered an EF of 10 mV/mm at 16 Hz in the form of degenerate sine waves to a human osteosarcoma cell line SaoS-2 cultured on microscopic coverslips in a glass Petri dish. Electric current was conducted into the chamber through calomel electrodes inserted in the synthetic rubber-agar bridges. The authors demonstrated that ES in the form of degenerate sine waves had a significant effect on osteoblast proliferation, differentiation, and mineralization.

Using salt bridges can separate the culture chamber from the harmful electrolytic products of the electrodes. However, the large diameter of the glass tube causes a potential problem of the salts leaking into the culture chamber and affecting the culture medium composition [34]. The experiment setup also occupies a large space, and the glass bridges may risk breaking inside a cell culture incubator. In the literature, there are many reports using salt bridges to deliver low voltage ES in vitro [35].

### 2.3. Working Electrode as Cell Culture Substrate

Under this configuration, cells are firstly seeded and cultured on the surface of an electrode, which then is used as the working electrode in a three-electrode electrochemical cell. As the surface of a working electrode has equipotential, all the cells adhered to it are subjected to a uniform EF (Figure 1C). Since cells behave differently on various substrates, any comparison between experiments involving different working electrodes must be cautious.

#### 2.3.1. Metal Electrode

The proliferation and morphology of human carcinoma cells MKN45 cultured on the surface of a platinum-coated plastic plate electrode were altered depending on the applied potential [36], with the cell proliferation halted when the potential was above 0.4 V vs. the reference electrode Ag/AgCl. Bieberich et al. [37] studied the synapse of the rat pheochromocytoma cells (PC12) and the blastocyst-derived murine embryonic stem cells (ES-J1) cultured on the interdigitated microelectrode arrays made of Au, Pt, or indium tin oxide (ITO). A sine wave of 100 mV peek-to-peek voltage at a frequency of 4000 Hz was applied. The PC12 and ES-J1 cells were either differentiated by incubation with nerve growth factors (NGF) and neuroactive drugs or cultured under serum deprivation and treatment with basic fibroblast growth factors (FGF-2). Interestingly, the authors found that the neuronal cells extended to form pili-like contacts on the surface of the ITO electrodes, implying the growth cone formation. Serena et al. [2] cultured human embryonic stem cells (hESCs) on electrodes of different metals, including stainless steel, titanium-nitride-coated titanium, and titanium. The cells were stimulated at 1.0 V/mm. It was found that the ES played a role in the cardiac cell differentiation of hESCs through the mechanisms associated with the intracellular generation of reactive oxygen species. The authors confirmed that the reactive oxygen species acted as intracellular secondary messengers and activated the signaling cascades involved in cell growth and differentiation. Gittens et al. [38] reported that osteoblast (MG63) differentiation and the production of factors such as osteoprotegerin, an inhibitor of osteoclastogenesis, and vascular endothelial growth factor, a potent angiogenic factor important for bone development, were enhanced by the electrically polarized titanium surface, with the polarizations ranging from 100 to 500 mV. Newbold et al. [39] investigated how charge-balanced biphasic pulsatile waveforms affected the Madin-Darby canine kidney cells cultured on a gold electrode. They used a train of pulses that were 50 µs width separated by 25 µs interphase gaps at a frequency of 200 pps. The current amplitudes were 0.5, 0.2, and 0.05 mA, corresponding to charge densities of 50, 20, and 5 µC/cm^2^, respectively. They found that a significant and rapid drop in both electrode impedance and access resistance occurred, and the magnitude of the impedance drop was linearly dependent on the current amplitude. Because the cells began to lose from the electrode surface immediately at the beginning of the stimulation, the authors believed that stimulation intensity caused localized changes in cell adhesion.

When cells are cultured on a metallic working electrode, they are not only exposed to EF but also to potential redox reactions. Therefore, one must be careful to avoid such unwanted interference. On the other hand, metal electrodes can be easily fabricated into complex configurations to accommodate varieties of requirements.

#### 2.3.2. Indium Tin Oxide (ITO) Electrode

As an important translucent conducting metal oxide with a wide band-gap of 3.55-3.75 eV [40], ITO has attracted a considerable attention due to its high electron affinity, low effective electron mass, low resistivity, high transparency in visible light region, good adherence to substrates, as well as high chemical stability [41].

Suzuki et al. [42] cultured hybridoma cells on a transparent electrode made of an ITO coated glass plate for the first time. They exposed the cells to alternating rectangular electric pulses at 5 kHz for 30 s followed by a 4.5 min pause, with a platinum mesh as the counter electrode. They found that the metabolic activities of the cells including monoclonal antibody production and reactive oxygen species such as superoxide and hydrogen peroxide were increased because of the ES. HeLa cells [43] were also tested on the ITO coated electrode and stimulated between 0.5 and 0.7 V. The cells were found viable but accompanied with morphological changes, from original spindle shape to round shape. Through photolithography, Qiu et al. [44] created two pairs of 13 mm × 76 mm parallel ITO electrodes on a glass slide with a 2.7 mm gap. After culturing rat bone marrow stromal cells and having them stimulated at 0.7, 0.8, 0.9, and 1.0 V, they found that the positively charged surface enhanced cell attachment but suppressed cell spreading and differentiation. They attributed these findings to the negatively charged cell membrane. Even in the absence of NGF, the differentiation of PC12 cells were induced to grow neurites by ES at amplitudes of 200 and 400 mV at frequencies of 50, 500, and 1 kHz [3].

When mouse astroglial cells and 3T3-L1 cells were cultured on an ITO electrode, it was found that the electric potential in sine waves enhanced the Hsp70 gene expression [5]. Tandon et al. [45] fabricated an ES system made of an interdigitated microarray of ITO electrodes to study microscale cell culture. These electrodes were 180 µm in width with 200 µm spacing. Primary cardiomyocytes and human adipose-derived stem cells were cultured on the electrodes and then experienced a pulsed ES of 2 ms duration at 1 Hz. Both cell types recorded an enhanced proliferation, elongated morphology, cell alignment, and a higher number of connexin-43-composed gap junctions.

Takayama et al. [46] developed microcavity-array patterns and fabricated the substrate with ITO through standard photolithography techniques. The embryoid bodies of mouse P19 cells were inserted in the microcavities and stimulated under a pulsed EF of 1000 V/m in strength and 1 ms in pulse wide. They reported that the ES induced specific spatiotemporal patterns of intracellular calcium transients.

Kajiya et al. [47] studied the behaviors of human dermal lymphatic endothelial cells (LECs) on two electrode materials. In one system, the cells were plated on a fibronectin-coated ITO electrode, with a platinum electrode placed above the cells to form capacitors. ES induced an extension of the actin filaments of the LECs, increased calcium influx, and phosphorylation of the p38 mitogen-activated protein kinase (MAPK). In another system, they plated the cells onto two ITO electrodes with a 0.66 mm gap in between and delivered ES from side to side. They reported that the LECs significantly migrated towards the cathode due to the pulsed-DC stimulation. Tanamoto et al. [48] cultured PC12 cells on ITO electrode and found that the efficiency of ES depended on electrode size and electric current.

A thin film of ITO can be deposited on a variety of substrates using numerous physical or chemical methods, such as electron beam evaporation, physical or chemical vapor deposition, sputtering coating, and sol-gel techniques [41]. ITO offers the possibility to investigate the cellular behaviors in EF in situ because of its conductive and transparent features. On the other hand, ITO is normally used on 2D substrates, not suitable for porous 3D scaffolds.

#### 2.3.3. Conducting Polymer Electrode

Wallace et al. [49] prepared a conductive composite made of polypyrrole (PPy) and poly(2-methoxy-5 aniline sulfonic acid). This material was used as a cell culture substrate in the form of two narrowly separated electrodes in a two-electrode system. They reported that charge-balanced biphasic electric pulses at 10, 100, and 250 Hz significantly promoted nerve cell differentiation in the presence of NGF. In another report, Wallace et al. [50] reported a 3D fibrillar conductive matrix made of the chondroitin sulfate doped PPy and collagen type I. They demonstrated that this 3D matrix improved the integration of the neural electrode with the nerve cells by promoting nerve cell attachment and differentiation under the biphasic pulsed ES at 250 Hz and 50 mV. By electrochemical polymerization, Sirivisoot et al. [51] loaded antibiotics (penicillin/streptomycin, P/S) and an anti-inflammatory drug (dexamethasone, Dex) to PPy, forming the PPy[P/S]- and PPy[Dex]-coated electrodes. Using a cyclic voltammetry scanning between −1 and 1 V at a rate of 100 mV/s, the authors reported the “on demand” release of P/S and Dex.

Hsiao et al. [52] cultured PC12 on poly(3, 4-ethylenedioxythiophene) (PEDOT) that was electrochemically coated on ITO electrodes. After applying an EF of 120 mV/mm, they found that neurites aligned along the direction of the photoetch patterned electrodes, and that the outgrowth of neurites was also promoted by the ES. Similarly, Zhu et al. [53] plated PC12, NIH3T3, and Schwann cells on the PEDOT-coated ITO electrodes to deliver electrical signals to the cells. The electrical signals were the charge-balanced biphasic ES at 250 Hz and 20, 40, or 60 mV. They reported that the ES greatly enhanced neurite outgrowth and promoted protein secretions from the Schwann cells.

Schmidt et al. [54] synthesized a PPy film with a resistance of approximately 1 kΩ. They used this PPy film as the anode and a gold (Au) wire placed at the opposite end of the culture well as the cathode. After delivering a steady 100 mV potential to the PC12 cells cultured on the PPy film, they found that the ES significantly increased neurite length. The authors concluded that the constant current passing the PPy membrane rather than the ionic current passing medium affected the cells. They also reported that ES increased fibronectin adsorption to PPy and thereafter might have enhanced PC12 neurite outgrowth [55].

## 3. Conductive Substrate Mediated ES

**Figure 2:**
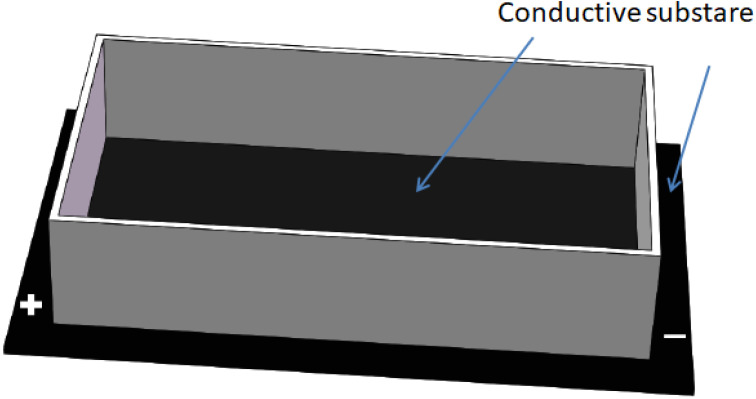
Conductive substrate mediated ES where the cells are cultured on the substrate and exposed to the potential gradient on substrate surface.

Different from being used as an electrode, a conductor or more likely, a semiconductor can be used as a substrate to grow cells (Figure 2). Such a substrate is wired in a complete circuit and does not generate any electrophoresis or electrode reaction. When a potential gradient is formed along the substrate, the cell membrane that intimately interacts with the substrate surface also experiences that potential gradient. Should an alternative current be used, the electromagnetic field would also play a role. The conductivity of the substrate must be low enough to enable a physiologically significant potential gradient and at the same time not too high to generate excessive Joule heat disturbing normal physiological temperature.

### 3.1. Carbon and Its Allotropic Substances

Carbon shows a high corrosion resistance and inertness under a wide variety of conditions, such as in strong acids and bases. Its surface can be modified by physisorption or chemisorption of the molecules of interest, or through immobilization of the recognition elements and enzymes [56]. Moreover, the carbon surface can offer better affinity than Pt or Au for carbon- and hydrocarbon-based materials. Thus, carbon is still widely used as an electrode material in electrochemistry.

Supronowicz et al. [57] prepared a conductive composite by blending poly(lactic acid) and carbon nanotubes (CNT) and used it to expose cells to ES. In their study, osteoblasts were cultured on this conductive composite, over which a stainless-steel electrode was immersed in the culture media 0.5 cm away from the cells. After exposing the cells to a 10 µA stimulation at 10 Hz for 6 h/day in various days, a 46% increase in cell proliferation after 2 days, a 307% increase in extracellular calcium after 21 days, and an upregulation of collagen type-I mRNA expression after both 1 and 21 days were recorded. When tungsten and stainless-steel wire electrodes were coated with CNT, the electrode impedance decreased and the charge transfer increased [58]. As a result, a CNT coating was found to enhance the effect of ES to neurons in culture. CNT was also mixed with laminin to mediate both the differentiation of and the ES to neural stem cells (NSCs) [59]. Cho et al. [60] prepared an electrically conductive composite made of collagen and CNTs as a substrate for the in vitro growth of PC12 cells, which were exposed to a constant voltage of 0.1 V for 6 h. They found a greater neurite and filopodium extension because of the better differentiation of PC12 cells into neurons. Shao et al. [61] prepared electrically conductive nanofibers with biodegradable poly-DL-lactide (PLA) and multiwalled CNTs. Using a constant current at 50, 100, and 200 mA, they found that osteoblasts grew along the electric current direction, and the topographical features of the substrate played a minor role. Mooney et al. [62] plated mesenchymal stem cells onto the surface of a conductive PLA/CNT composite and exposed them to the electric pulses of 0.15 V/cm intensity and 2 ms wide at 1 Hz in a home-made electrophysiological bioreactor. After stimulation, the cells were found reoriented perpendicular to the direction of the current and adopted an elongated morphology; more interestingly, there was evidence showing an increased cross-talk between the cells. Stout et al. [63] seeded rat aortic endothelial cells, fibroblasts, and cardiomyocytes onto the poly(lactic-co-glycolic acid)/carbon nanofiber composites, and exposed the cells to a continuous ES (rectangle, 2 nm, 5 V/cm, 1 Hz) through a commercial C-Pace EM device. They reported a minor change in cardiomyocytes and a hindered proliferation in endothelial cells and fibroblasts. Meng [64] compared the constant (100 mV/mm) and programmed ES (100 mV/mm at 1 and 10 Hz) to PC12 cells cultured on a non-functional graphene nano-film and found that only the programmed ES enhanced PC12 cell differentiation, neurite extension, and growth. Although carbon and its allotropic substances have already presented broad compatibility to in vitro ES experiments, some disadvantages still exist, such as variations in quality and difficulties in shaping. Up until recently, as well, there were still arguments that carbon might have cytotoxicity to some extent [65].

### 3.2. Conducting Polymers

**Figure 3:**
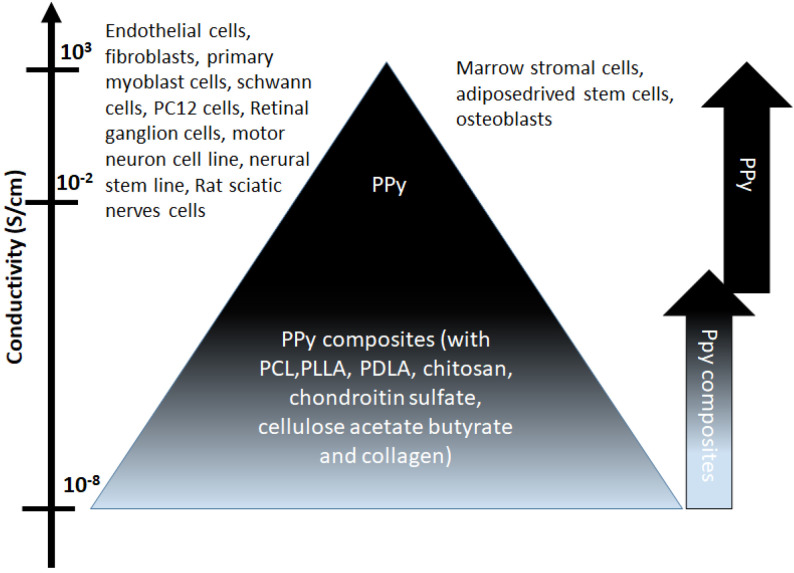
Cells and PPy or its composite served as electrodes and substrates used for electrical stimulation. PPy sheets or PPy membranes synthesized by electrochemical/chemical methods present higher conductivity compared to the PPy composites. (PCL: polycaprolactone, PLLA: poly-L-lactide, PDLA: poly-D-L-lactide).

Due to its rigid backbone of the π-conjugated structure, conducting polymer PPy exhibits poor mechanical properties. Different approaches have been reported to prepare its composites to overcome this limitation (Figure 3). Shi et al. reported a biodegradable conductive composite made of PPy nanoparticles and poly(D,L-lactide) (PLLA), and stimulated human dermal fibroblasts with various ES intensities ranging from 50 to 100 mV/mm [66,67]. They demonstrated an increased cell growth and a change in cytokine secretion. Jeong et al. (2008) reported an electrospun matrix made of polyaniline and poly(L-lactide-co-epsilon-caprolactone). They found that a 20 mA DC significantly increased the mitochondria metabolic activity of the NIH-3T3 cells. By delivering a 200 mV/mm ES through the conductive PLLA/PPy membranes to SaoS-2 osteoblasts, Meng et al. [64,68,69] demonstrated that the ES significantly upregulated the expression of the osteoblast-specific markers ALP, BMP2, Runx2, and OC. Human skin fibroblasts cultured on the PPy/PLLA membranes in an EF of 200 mV/mm showed higher migration and proliferation rates, upregulated secretion of fibroblast growth factor 1 (FGF1), and FGF2, and augmented transdifferentiation into myofibroblasts [69]. In 2013, Zhang et al. [70] cultured osteoblast MC3T3-E1 on BMP-2-functionalized PPy/chitosan films subjected to a 200 µA DC stimulation. They found that the ES increased cellular metabolic activity, ALP activity, and mineralization. Schmidt’s group (Durgam et al., 2010) synthesized a copolymer of PPy and PCL, which is both conductive and biodegradable.

On the other hand, by coating PPy directly on other supporting polymers, a conducting PPy layer can be prepared to serve as conductive substrates. Zhang et al. [9] reported that DC ES enhanced PC12 neurite outgrowth on the microporous surface of a PPy-coated poly(D,L-lactide-co-epsilon-caprolactone) (PDLLA/CL) membrane. Using a scaffold made of polycaprolactone fumarate and coated with PPy, Moroder et al. [71] stimulated cultured PC12 with either a constant DC of 10 μA (7.2 μA/cm^2^) or a train of square pulse of the same current intensity but at a 20 Hz frequency with the pulse width being half the interval. The authors found that the two types of ES resulted in a similar percent of the neurite bearing cells and a similar neurite length and that the neurites aligned parallel to the direction of the applied current. Cho et al. [72] prepared a porous conducting PPy film loaded with nerve growth factors (NGF) by the electrochemical deposition of a mixture of pyrrole monomers and NGF into two- or three-dimensional particle arrays. After applying a constant electrical voltage of 0.1 V/mm for 2 h, they reported that both the surface topography and the EF played a crucial role in determining cell responses, including enhanced cellular viability and neurite extension. Hu et al. [73] coated PPy onto the polystyrene Petri dish to form a semitransparent thin film with 1.0 - 10^−3^ S/cm conductivity. They then used a constant EF to study the effect of ES on the osteogenesis of rat bone marrow stromal cells. They found that an ES at 0.35 and 0.035 V/cm improved calcium deposition in the extracellular matrix resulting in higher levels of mineralization. Cell death occurred at 3.5 V/cm together with the appearance of bubbles that might be caused by the high voltage, as suggested by the authors. Unfortunately, the authors did not mention the nature of the bubbles.

Using PPy-coated flexible poly(ethylene terephthalate) (PET) textile as an ES vehicle, freshly harvested rat sciatic nerves were exposed to a 160 mV/mm (conductive channels) or 200 mV/mm (inductive channels) pulsed ES at 2 Hz for 48 h. Du et al. [74] designed two ex vivo tissue culture models to mimic the ES applied through nerve channels. The nerve tissue had the original architecture and cell populations. It was found that the ES accelerated Wallerian degeneration, helped Schwann cells to switch into a migratory phenotype, and upregulated the secretion of multiple neurotrophic factors. The EF also induced cell migration inside the axon.

In addition to PPy, PEDOT is also widely studied, mostly in the area of neural implants. PEDOT exhibits a high transparency and a satisfactory chemical stability. A PEDOT:PSS suspension is also commercially available for surface treatment. Pires et al. [75] cultured neural stem cells on PEDOT:PSS, then a pulsed DC EF (1 V/cm, 100 Hz, 10 ms pulses) was applied horizontally along the substrate by two parallel strips of gold outside of the culture well. They reported that the ES enhanced neural stem cells differentiation and neuron elongation aspect ratio as well as neurite length.

Conductive polymers can become biocompatible, biodegradable, and porous after appropriate structure design and manufacturing processes. For example, by incorporating antibodies, enzymes, or other biological moieties, conductive polymers’ chemical and physical properties can be modified accordingly [76]. Through a template-assisted interfacial polymerization (TIP) technique using methyl orange as the template, Mao et al. [77] synthesized a truly soft pristine 3D PPy membrane that can maintain high flexibility even in liquid nitrogen. Such a membrane can be folded into various shapes such as boxes and tubes. This PPy membrane showed a tensile strength of 90 kPa at 2.5% elongation at break and an elastic modulus of 3.4 MPa at 1% strain, with a conductivity at 1.5 S/cm, which is high for the chemically synthesized PPy. This soft pristine 3D and large-sized PPy membrane might pave the way for practical applications.

## 4. Noninvasive Stimulation

### 4.1. Electromagnetic Field

**Figure 4:**
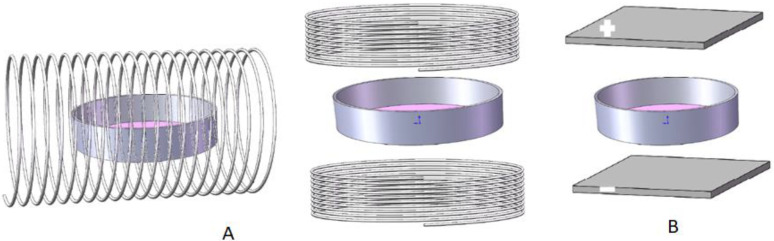
Two designs offering noninvasive ES. (**A**) ES through EMF, where a cell culture plate is placed inside a Helmholtz coil in parallel to the direction of MF, or in perpendicular to the direction of MF between two Helmholtz coils; (**B**) ES through capacitive coupling where a culture plate is sandwiched between two paralleled plates (note: the distance between plates and culture should be minimum to reduce resistance).

A noninvasive stimulation can be realized through an electromagnetic field (EMF) (Figure 4A). To perform such an experiment, the working coils generating EMF surround or encase the study subject without any direct contact. Evidently, EMF stimulation bypasses some important issues inherently related to electrodes, such as cytotoxicity, tissue compatibility, charge transfer, electrode surface modification, and corrosion.

Evidence [78] supporting the effect of the extremely low-frequency (ELF) sinusoidal signals at the cellular level demonstrated that EMF could have dramatic effects on ligament cells. The authors suggested the usefulness of such a bioelectrical stimulation for the growth and repair of damaged tissues. In the laboratory, a uniform EMF can be easily generated inside a pair of Helmholtz coil. Low-frequency electric fields (LFE) do not penetrate cells very effectively because of the low dielectric constant of the cell membrane; however, low-frequency magnetic fields do penetrate. Electric and magnetic fields act at different sites and by different mechanisms. EFs affect charge distribution at interfaces and penetrate the interior by polarizing. On the other hand, magnetic fields affect mobile charges throughout a tissue [79].

To confirm if ELF-EMF would cause cell apoptosis and disturb the cell cycle of different cell lines, Martinez Morillo et al. [80] exposed two cell lines (U-937 and HCA-2/1 cch,) to a 25 Hz and 1.5 mT EMF generated through two 15 cm × 10.5 cm air core solenoids. The cell cycle, apoptosis (spontaneous and dexamethasone-induced), and growth were evaluated. However, this work showed neither a significant alteration in cell cycle phases nor an induction of apoptosis. Nevertheless, the authors noticed that the relative cell number decreased to 55.84% ± 7.35% following the exposure to EMF, which however, could be due to the presence of dexamethasone. Ding et al. [81] exposed PC12 cells to a pulsed EMF at 1.36 mT (peak value) generated by a pair of 13 cm radius Helmholtz coils (each made of 300 turns of enameled copper wire with 0.4 mm radius). In their experiment, duty cycles of 10%, 30%, 50%, 80%, and 100% (DC) were used. They found that the EMF generated by narrow (10%) pulses significantly reduced the number of the neurite bearing cells and the number of neurites extended along the direction of the EMF, but increased the average length of the neurites. On the other hand, an 100% duty cycle (equivalent to DC) had exactly the opposite effects. Partridge et al. [82] cultured rat primary osteoblasts between a pair of 29.5 cm diameter Helmholtz coils generating triangular pulses of EMF (pulse frequency 3.8 kHz, burst frequency 1.5 Hz, burst duration 25.6 ms, and burst period 670 ms). They found that the pulsed EMF increased ALP mRNA expression. When chondrocytes were exposed to a 0.6 T static magnetic field (SMF) and a pulsed EMF (21.2 MHz, pulse period 15 ms, burst duration 2 ms, amplification 3 dBm (0.1 V) and maximum output 250 W) respectively, Sabo et al. [83] found that the SMF resulted in a statistically significant increase in cell viability but the pulsed EMF did not.

Chang et al. [84] generated EMFs of 0.13, 0.24, and 0.32 mT by passing 7.5 Hz pulses of 300 μs wide through the coils. This was translated into induced EFs of 2, 4, and 8 mV/cm in culture medium. The authors found that the EMF stimulated the growth of osteoblasts, release of TGFβ1, and increase in ALP activity. In 2009, the same group [85] exposed human mesenchymal stem cells to a daily EMF stimulation at 0.13 mT corresponding to an induced EF of 2 mV/cm. Both cell proliferation and osteogenic gene (Runx2/Cbfa1 and ALP) expression were found significantly enhanced. In Nikoshkov’s experiments [86], cultured human dermal fibroblasts were maintained in a very weak 1 GHz EMF of 5 nW/cm^2^ generated through a conical antenna connected to the Aquaton-2 generator. The authors reported that such a super weak high-frequency EMF could activate fibroblast migration and proliferation and suggested that this was probably because of the oscillation of water molecules in EMF. Saito et al. [87] developed a localized magnetic stimulation method to stimulate the cultured neuronal cells by placing a ring made of Mu-metal over the cells. They also performed a numerical simulation to demonstrate the localization of the field. They found that the localized magnetic stimulation inhibited the synchronized periodic bursting of the cortical neuronal network, and such an effect was lasting at a higher frequency.

Mihai et al. [88] studied the behaviors of Vero cells under the influence of EMF. The pulsed 100 Hz EMF was generated with a pair of Helmholtz coils of 29 cm in diameter and made of 620 turns of a copper wire, assuring a homogeneous 5.6 mT magnetic field in the center of the coils. After being exposed to EMF, the Vero cells recorded an increase in the number of cells together with a high percentage of damaged DNA. Quantitative evaluation of the DNA damage using comet assay showed the increases in the tail length, the quantity of DNA in the tail, and the olive tail moment. Cell cycle analysis showed a high frequency of the cells in the S phase, supporting single strand breaks. They thought that the reason could be the production of different types of reactive oxygen species. With a solenoid composed of three series-connected coils winding on an acrylic cylinder of 18 cm inner diameter and 21 cm long, which was capable of producing a magnetic flux density in the range of 0.0–9.0 mT at 5–200 Hz frequencies, Chen et al. [89] reported that the waveforms (sinusoidal, triangular, square, and serrated, all set at 50 Hz and 1.8 mT intensity) of EMF played a key role in the proliferation, differentiation, and mineralization of rat calvarial osteoblasts. Specifically, the square EMF promoted the proliferation but had no effect on the differentiation of the osteoblasts; the sinusoidal EMF inhibited cell proliferation but enhanced osteogenic differentiation, and the triangular EMF did not affect cell proliferation but induced the strongest osteogenic activity.

Ho et al. [90] developed an EMF generating system that could generate pulses of 5 ms width in the form of sine waves, with adjustable frequency and intensity. They studied the behaviors of human bone marrow mesenchymal stem cells and found that treatment of 30 pulses at 1 T for five days was not cytotoxic. They reported the increased proliferation, osteogenic differentiation, and mineralization of the cells in vitro and improved bone healing in vivo. What is very interesting in this study is that the authors demonstrated that short time and high intensity could be a good combination. Using pulsed EMFs of 1, 2, and 5 mT with a modulation frequency at 750 Hz and a carrier frequency at 75 Hz, a duty ratio of 0.8 and a stimulation period of 3 h/day for four weeks, generated between two Helmholtz coils, Tan et al. [91] found that the pulsed EMFs at all the three intensities inhibited cartilaginous phenotype and degraded cartilage-specific extracellular matrix in chondrogenic differentiation. However, a pulsed EMF at 1 mT directed the differentiation of the chondrogenic-induced stem cells to the hypertrophic stage and promoted the osteogenic differentiation. Chen et al. [92] exposed osteoclast-like cells to triangular waves of a pulsed EMF at 8 Hz and 3.8 mT inside a cylindrical solenoid made of 1000 turns of a 1 mm diameter copper wire. They reported that the EMF might have modulated osteoclast activation hence bone resorption, probably through the expression of nuclear factor of activated T cells c1 (NFATc1) and carbonic anhydrase isoenzyme II (CAII).

EMF was also found to stress cells [88,93,94], and such stress response does not occur in all types of cells, knowing that even the same cell line may give opposite results in the same laboratory [95]. On the other hand, an EMF is still not spatially well-defined and alternates over time. Therefore, the localization and discrimination of the stimulated area remain difficult even though a localized magnetic stimulation system has been reported [87]. Stimulation with EMF makes the ES experiment simplified as no electrodes are required. However, compared with the electrode-based methods, it brings another factor into the ES system: the magnetic field. Therefore, it is hard to tell whether the stimulation effects on the cells could be specifically attributed to the EF, magnetic field, or both.

### 4.2. Stimulation through Capacitive Coupling

ES through capacitive coupling is based on the EF established between two capacitor plates, between which the cells or tissues to be stimulated are sandwiched (Figure 4B). To ensure a uniform ES, parallel plates are normally used. The strength of the EF applied to the cells or tissues can be calculated if the dielectric constants of the materials between the capacitor plates are known.

In 1984, Korenstein et al. [96] set two 54 mm diameter parallel copper plates above and below a Petri dish where bone cells isolated from rat embryo calvaria were cultured. A series of rectangular pulses of 25 µs width at 3 Hz were generated under voltages from 10 to 54 V/cm, resulting in an instantaneous displacement current of 0.7–4.0 A in culture medium. They found that such a capacitive ES also caused changes in intracellular cyclic AMP and enhanced DNA synthesis. It is important to be aware that the voltages mentioned above were not the EF strength in culture medium, but the ones applied to the two copper plates.

Since 1984, Brighton’s group has used capacitive ES to treat different cell types. The difference between their setup and Korenstein’s is that the Petri dish is placed between stainless steel capacitor plates without an air gap between the plates and the dish. In such a way, one requires a significantly lower voltage to induce the same EF with respect to the setup having an air gap. By studying the behaviors of articular cartilage chondrocytes under the influence of the capacitively coupled 60 kHz sine waves of 10, 100, 250, and 1000 V peak-to-peak potential gradient for 24 h, Brighton et al. demonstrated that the capacitive ES increased cell proliferation and the synthesis of glycosaminoglycans [97]. In 1985 [98], the authors exposed fetal rat tibiae to a capacitively coupled ES in the form of 60 kHz sine waves with a 10 V peak-to-peak output, which should have induced a current intensity of 5.2 µA/cm^2^ and a field strength of 0.32 mV/cm in the medium. They reported that the EF inhibited the oxygen-induced osteoporosis.

In a series of experiments, Armstrong et al. (1988) studied bovine chondrocytes subjected to various capacitively coupled EFs generated under 0, 10, 100, 250, 500, 750, 1000, and 1500 peak-to-peak output voltages at 60 kHz, which theoretically should have induced the EF from 1.5 to 4.5 × 10^−2^ V/cm. The authors recorded a significantly increased cell proliferation at 500, 750, and 1000 V, supported by an increased cAMP production. However, the cell proliferation was significantly inhibited at 1500 V or 4.5 × 10^−2^ V/cm. They also exposed fetal rat calvarial bone cells to a capacitively coupled EF of 2.62 mV/cm for 2.5–30 min and found reduced cAMP synthesis.

In 1989, Brighton et al. [99] reported that the biologic response of bovine growth plate chondrocytes in vitro was signal specific. The total amount of electric energy required for chondrocyte growth was as small as a 3.6 min ES in 24 h using a 0.25% duty cycle under the ES intensity of 7, 20, 50, and 126 mV/cm. In 1992, Brighton et al. [100] reported that field strength (0.1, 1, and 20 mV/cm) played a dominant role in determining the proliferative response of rat calvarial bone cells and the alkaline phosphatase activity. Rat calvarial bone cells and mouse MC3T3-E1 cells [101] were subjected to a 20 mV/cm ES, showing a significant enhancement in cell proliferation determined by the deoxyribonucleic acid content. The authors also claimed that the capacitively coupled field stimulated the transmembrane calcium translocation via various processes, including voltage-gated calcium channels, the activation of phospholipase A2, a subsequent increase in prostaglandin E2, as well as the increase in cytosolic calcium and activated calmodulin. In 2001, Brighton et al. [102] reported that capacitive coupling could induce Ca^2+^ translocation through the cell membrane voltage-gated calcium channels, resulting in increased cytosolic Ca^2+^ and activated cytoskeletal calmodulin. In 2014, this group [103] delivered the capacitively coupled EF of 60 kHz, 20 mV/cm, 50% duty cycle for 2 h per day, to human calvarial osteoblasts. They found that the expression of several genes was significantly upregulated, including the transforming growth factor TGF-β family genes (BMP-2 and -4, TGF-β1, -β2 and -β3) as well as the genes of fibroblast growth factor FGF-2, osteocalcin, and ALP. The protein levels of BMP-2/4 and TGF-β1/β2 were also increased. In Bayat’s design [33], the upper plate was placed above the medium, leaving an air gap of ~2 mm, and the total separation between the plates was ~7.7 mm. Using an EF of 10 mV/mm and 16 Hz, they reported the increase in collagen I, osteocalcin, and osteonectin from osteoblast-like cells SaOS-2.

Jain et al. [104] pyrolyzed polyacrylonitrile coated on a silicon wafer to produce a pyrolytic carbon with a high specific charge storage capacity (0.2 mC/cm^2^) and a low impedance (3.3 k ohm at 1 kHz). They used such a porous carbon surface as substrate to grow mouse neuroblastoma cells and then formed a capacitive EF between the carbon substrate and a stainless-steel plate placed above the culture medium, either parallel or inclined with respect to the pyrolytic carbon surface. This design was to void the inherent drawbacks associated with the submerged electrode, such as electrode corrosion or ionic current. The authors found that the cell viability and neurite length were higher at the low EF strength (≤2.5 V/cm).

Capacitive plates can effectively deliver ES to cultured cells or tissues in a noninvasive way. Specifically, this method avoids any contact between cells and capacitive plates, eliminating the potential interference of the surface chemistry and morphology of the plates. However, due to the dielectric between the capacitive plates and cells, a high (may excess 1000 volts) and unsafe voltage is required.

## 5. Conclusions

Electrodes made of various materials are the most popular methodology to deliver ES. Customized devices modified from commercial Petri dishes or flasks were used to host the electrodes that were either directly immersed in culture medium or mediated by salt bridges. Precious metals such as silver is often selected due to the relative ease in fabrication; however, they are very unstable over time [105]. The charge injection capacity of some metals such as Pt and Ir can be enhanced with the help of their oxidation. Pt offers a more stable noise and an additional storage of charge at the electrode surface [106].

To avoid the corrosion of metals, titanium nitride, carbon, or conducting polymers are often used [107]. Compared to metals, carbon or hydrocarbon coatings represent relatively soft and biocompatible platforms. However, ES may change the oxidation state of conductive polymers by driving counterions out of the material, which might negatively impact the fast and efficient charge injection into the conducting polymers [108]. Carbon and transparent conductive oxides might age, showing small yet measurable changes in electrode properties after just one week of stimulation [45]. As far as salt bridges are concerned, they bridge the ionic flow between electrodes and the cell culture chamber and ensure minimum disturbance to the culture medium. However, the leaking of salts into the culture medium is still an issue that should be cautiously observed.

Organic conductive materials can be fabricated into different structures such as sutures, tubes, sheets, and porous scaffolds, opening a wide possibility of the electric manipulation of cells in 3D biomimetic systems. There is also an increasing interest in employing conducting polymers as a soft and biocompatible conductive interface in neural prostheses or as part of the bioelectronic devices. One advantage is that conductive polymers can be chemically or electrochemically modified to combine with biomolecules to form bioactive surfaces. CNTs and graphene or their composites also have potentials similar to conductive polymers; however, the cytotoxicity of such materials remains a major concern [109].

Most customized setups reported in the literature have a large profile and occupy a significant space inside and outside of the cell culture incubator (Table 1). Advanced technologies can help to miniaturize the devices and at the same time make them more robust. The most critical and complex factor in any ES experiment is what type of electric signals are delivered, to which cells will respond differently. These signals vary in DC or AC, field intensity and orientation, frequency and waveform, duty cycles, etc., compounded by the variety of setups and the co-existence of EF and MF. Theoretical analyses and precise measurements of the EFs at the substrate–cell interface of these setups are critical to further advance this field [109]. Finally, developing a few “standard” ES methodologies that permit a meaningful comparison across platforms is one of the biggest challenges.

## Figures and Tables

**Table 1:** Electrical stimulation regulated cells and cellular behaviors.

The Main ES	Method	Cells	Cellular Behavior Regulation	Refs.
Electrode-based ES	Two electrodes immersed in culture medium	Nerve cells, rat fracture callus cells, human peripheral blood lymphocytes, lymphoid cells, cardiac cells, human mesenchymal stem cells.	Increase cell growth, migration, proliferation and differentiation. For example, enhanced outgrowth of nerve fibers, higher neuron survival rate, and cell migration; uptake DNA precursors.	[11,12,18,20]
	Using salt bridges between electrodes	Neuronal cell, primary osteoclasts and osteoblast-like cells, fibroblasts, neuroblastoma cells, epithelial cells, bone marrow-derived mesenchymal stem cells.	Modify the adhesion, orientation and migration of cell differentiation and growth. For example, different cells migrate to different electrodes.	[13,23,24,26,27,28,29]
	Working electrodes as cell culture substrate	Rat pheochromocytoma cells and the blastocyst-derived murine embryonic stem cells, human embryonic stem cells, osteoblast, astroglial cells, primary cardiomyocytes and human adipose-derived stem cells, human dermal lymphatic endothelial cells.	Enhance proliferation, elongated morphology, cell alignment, activate the signaling cascades involved in cell growth and differentiation. For example, enhanced gene expression and increase superoxide and hydrogen peroxide.	[1,2,37,38,45,50,53,54,55]
Conductive substrate mediated ES	Carbone and its allotropic, and conducting polymers	Neural stem cells, mesenchymal stem cells, endothelial cells, fibroblasts, and cardiomyocytes, osteoblasts.	Increase cell growth, migration, proliferation and differentiation. For example, cytokine secretion and gene expression.	[59,60,61,62,63,64,66,67,68,69,73,75,110,111]
Noninvasive ES	Electromagnetic field	Osteoblasts, human bone marrow mesenchymal stem cells, osteoclast-like cells.	Increase cell viability, played a key role in the proliferation, differentiation, and mineralization of rat calvarial osteoblasts. Modulate osteoclast activation hence bone resorption, probably through the expression of nuclear factor of activated T cells c1 (NFATc1) and carbonic anhydrase isoenzyme II (CAII).	[81,82,83,84,85,86,87,90,92]
	Capacity coupling	Bone cells, articular cartilage chondrocytes, mouse neuroblastoma cells.	Play a dominant role in determining the proliferative response of bone cells and the alkaline phosphatase activity, and up-regulate the expression of several genes.	[96,97,98,99,100,101,102,103,104]

## Data Availability

Data sharing is not applicable to this article.

## Abbreviations

AC: alternative current; CNT, carbon nanotubes; DC, direct current; ECM, extracellular matrix; EF, electric field; ELF, extremely low-frequency; EMF, electromagnetic field; MF, magnetic field; ES, electrical stimulation; GF, growth factor; ITO, indium tin oxide; PDMS, polydimethylsiloxane; PEDOT, poly(3,4-ethylenedioxythiophene); PPy, polypyrrole; PLLA, poly(D,L-lactide).
